# Sustained postsynaptic kainate receptor activation downregulates AMPA receptor surface expression and induces hippocampal LTD

**DOI:** 10.1016/j.isci.2021.103029

**Published:** 2021-08-25

**Authors:** Jithin D. Nair, Ellen Braksator, Busra P. Yucel, Alexandra Fletcher-Jones, Richard Seager, Jack R. Mellor, Zafar I. Bashir, Kevin A. Wilkinson, Jeremy M. Henley

**Affiliations:** 1 Centre for Synaptic Plasticity, School of Biochemistry, Centre for Synaptic Plasticity, Biomedical Sciences Building, University of Bristol, University Walk, Bristol BS8 1TD, UK; 2 Centre for Synaptic Plasticity, School of Physiology, Pharmacology and Neuroscience, Centre for Synaptic Plasticity, Biomedical Sciences Building, University of Bristol, University Walk, Bristol BS8 1TD, UK; 3 Centre for Neuroscience and Regenerative Medicine, Faculty of Science, University of Technology Sydney, Ultimo, NSW, Australia

**Keywords:** molecular neuroscience, cellular neuroscience

## Abstract

It is well established that long-term depression (LTD) can be initiated by either NMDA or mGluR activation. Here we report that sustained activation of GluK2 subunit-containing kainate receptors (KARs) leads to α-amino-3-hydroxy-5-methyl-4-isoxazolepropionic acid receptor (AMPAR) endocytosis and induces LTD of AMPARs (KAR-LTD_AMPAR_) in hippocampal neurons. The KAR-evoked loss of surface AMPARs is blocked by the ionotropic KAR inhibitor UBP 310 indicating that KAR-LTD_AMPAR_ requires KAR channel activity. Interestingly, however, blockade of PKC or PKA also reduces GluA2 surface expression and occludes the effect of KAR activation. In acute hippocampal slices, kainate application caused a significant loss of GluA2-containing AMPARs from synapses and long-lasting depression of AMPAR excitatory postsynaptic currents in CA1. These data, together with our previously reported KAR-LTP_AMPAR_, demonstrate that KARs can bidirectionally regulate synaptic AMPARs and synaptic plasticity via different signaling pathways.

## Highlights

• Sustained kainate receptor (KAR) activation downregulates surface AMPARs
• KAR ionotropic signaling induces AMPAR LTD (KAR-LTD_AMPAR_)
• KAR-LTD_AMPAR_ is dependent on the GluK2 KAR subunit and calcineurin

## Graphical abstract

**Figure:**
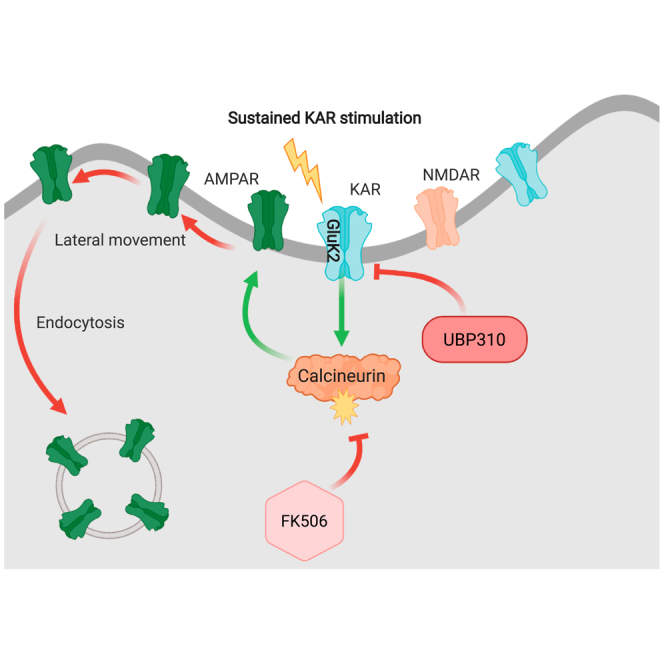

## Introduction

By activating distinct classes of both ionotropic and metabotropic receptors, glutamate mediates the overwhelming majority of excitatory neurotransmission in the mammalian central nervous system (CNS) and is critical for nearly all aspects of brain function. Ionotropic glutamate receptors comprise N-methyl-D-aspartate receptor (NMDAR), α-amino-3-hydroxy-5-methyl-4-isoxazolepropionic acid receptor (AMPAR), and kainate receptor (KAR) subtypes, whereas metabotropic glutamate receptors comprise 3 subfamilies of 8 mGluRs (groups I-III; mGluR1-8) (Collingridge et al., 2009).

Each of these receptor subtypes has been implicated in forms of synaptic plasticity that enhance or decrease the efficiency of synaptic transmission; long-term potentiation (LTP) and long-term depression (LTD), respectively. LTP and LTD occur at many different synapses across the CNS and are critical throughout life for processes ranging from synaptic formation and maturation during development, through to high level cognitive functioning and multiple aspects of learning and memory (Citri and Malenka, 2008; Malenka and Bear, 2004).

In part due to their fundamental roles in learning and memory, dysregulation of LTP and LTD is a prominent feature of cognitive decline in aging and a wide range of neurological and neurodegenerative disorders (Cuestas Torres and Cardenas, 2020; Kauer and Malenka, 2007). While the best characterized forms of LTP and LTD are initiated by activation of postsynaptic NMDARs (Collingridge et al., 2013; Hardingham, 2019), other induction pathways also exist. For example, there are mGluR-dependent forms of both LTP and LTD (Anwyl, 2009; Gladding et al., 2009), and more recently, it has been shown that activation of KARs can induce of LTP of AMPARs (Petrovic et al., 2017).

Regardless of the induction pathway, changes in the numbers and properties of surface-expressed postsynaptic AMPARs, and the consequent persistent strengthening or weakening of AMPAR-mediated transmission, underpin LTP and LTD (Henley and Wilkinson, 2016; Malenka and Nicoll, 1999). In summary, increases in surface-expressed synaptic AMPARs lead to functional and structural LTP, whereas decreases in surface-expressed synaptic AMPARs lead to LTD (Diering and Huganir, 2018; Huganir and Nicoll, 2013).

Compared with NMDARs and AMPARs, the functional roles of synaptic KARs have been enigmatic, but accumulating evidence demonstrates that KARs are key regulators of synaptic function (Evans et al., 2019). KARs can be expressed at both pre- and postsynaptic sites throughout the brain, where they contribute to the regulation of transmission, neuronal excitability, and network activity (Lerma and Marques, 2013). Intriguingly, in addition to operating as an ionotropic receptor, KARs can also signal through a non-canonical pertussis toxin-sensitive G protein-dependent pathway that is independent of ion flow through the channel (Melyan and Wheal, 2011; Rodriguez-Moreno and Sihra, 2007).

Previous work has demonstrated that relatively low level stimulation of KARs (10 μM KA for 3 min) in cultured neurons and hippocampal slices increases surface expression of KARs (Carta et al., 2013; Martin et al., 2008; Rivera et al., 2007; Selak et al., 2009) and leads to spine growth mediated by changes in post-endocytic sorting and enhanced recycling (Gonzalez-Gonzalez and Henley, 2013). Building on these observations, we examined whether transient KAR activation also impacts on AMPARs. We found that metabotropic KAR signaling can promote AMPAR surface expression and increases both AMPAR colocalization with PSD95 and AMPAR mEPSCs in hippocampal neurons and slices, revealing a physiologically relevant form of postsynaptic KAR-dependent, NMDAR-independent, LTP (KAR-LTP_AMPAR_) (Petrovic et al., 2017).

In contrast with transient KA stimulation, sustained stimulation of KARs (10 μM KA for 20 min) leads to their long-lasting loss from the cell surface (Gonzalez-Gonzalez and Henley, 2013; Martin et al., 2008; Martin and Henley, 2004). However, whether sustained KAR stimulation can also induce plasticity of AMPARs has not been investigated. Here we report a form of AMPAR LTD that can be induced by sustained stimulation of KARs (KAR-LTD_AMPAR_).

## Results

### Sustained KA treatment decreases surface levels of both AMPARs and KARs

**Figure 1:**
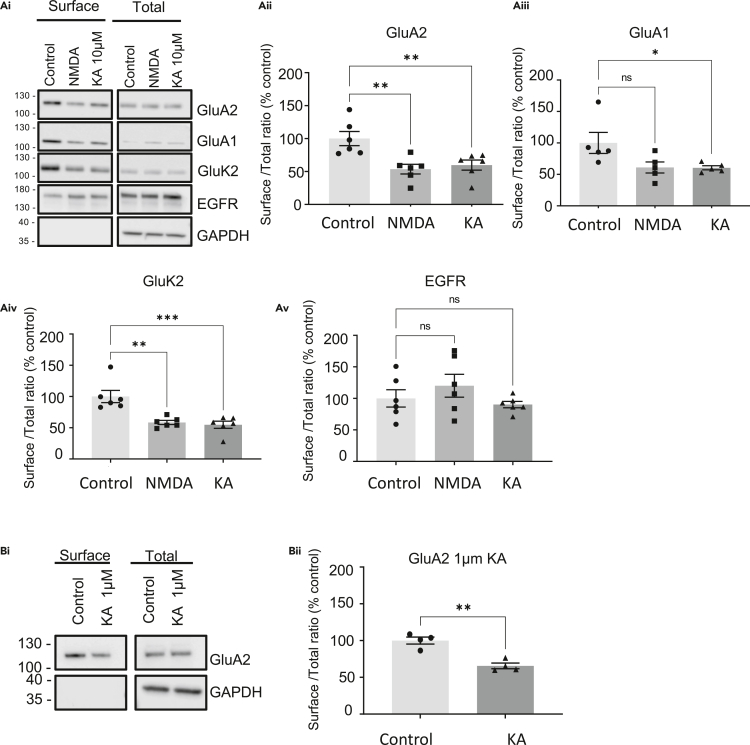
KAR activation reduces surface expression of AMPARs and KARs DIV 18 cultured hippocampal neurons were pre-treated for 30 min with 1 μM TTX and 40 μM GYKI 53655 before treatment with vehicle or 10 μM or 1 μM KA for 20 min. For NMDA treatment neurons were pre-treated with 1 μM TTX for 30 min followed by 3 min of treatment with 20 μM NMDA and 20 μM glycine. Surface proteins were biotin labeled and isolated by streptavidin pulldown, and lysates and surface fractions Western blotted. (A) (Ai) Representative Western blots of surface and total levels of GluA2, GluA1, GluK2, and EGFR. EGFR was used as a non-glutamate receptor expressed on the neuronal surface. GAPDH was used as a control to ensure no internal proteins were biotinylated. The surface to total ratio was calculated and expressed as a percentage of the control for (Aii) GluA2, (Aiii) GluA1, (Aiv) GluK2, and (Av) EGFR. N = 6 experiments from independent dissections, ∗p < 0.05, ∗∗p < 0.01, ∗∗∗p < 0.001; one-way ANOVA with Dunnett’s multiple comparisons test, error bars = SEM. (B) 1 μM KA effectively reduces surface expression of GluA2-containing AMPARs. (Bi) Representative Western blots of surface and total levels of GluA2. (Bii) Quantification of the surface to total ratio of GluA2 expressed as a percentage of control. N = 4 experiments from independent dissections, ∗∗p < 0.01, Un-paired t test with Welch’s correction, error bar = SEM.

Most AMPARs are heterotetramers of GluA1/GluA2 subunits or GluA2/GluA3 subunits (Zhao et al., 2019). Therefore, we first tested the effects of sustained KA application (10 μM KA for 20 min) on surface expression levels of the AMPAR subunits GluA1 and GluA2, and the KAR subunit GluK2, in cultured hippocampal neurons. Neurons were pre-treated for 30 min with 1 μM tetrodotoxin (TTX), to prevent depolarization-evoked presynaptic glutamate release, and 40 μM GYKI 53655, an AMPAR-specific antagonist (Partin and Mayer, 1996; Paternain et al., 1995), to prevent direct activation of AMPARs by KA. In parallel, on neurons from the same dissection, we used a well-established NMDAR-mediated chem-LTD protocol comprising 20 μM NMDA and 20 μM glycine for 3 min, followed by a 17-min incubation in the absence of NMDA, to allow receptor internalization (Ashby et al., 2004; Glebov et al., 2015; Lee et al., 1998). Surface proteins were then labeled by biotinylation and isolated by streptavidin pulldown. Surface proteins and whole-cell lysates (total protein) were analyzed by Western blotting for GluA2, GluA1, and GluK2 (Figure 1A). Surface expression of GluA2, GluA1, and GluK2 were all significantly reduced to similar levels by both KA and NMDA treatment. Importantly, these effects are selective since there was no change in the surface expression of EGFR, a non-iGluR protein used as a control.

KA is a partial, weakly desensitizing agonist at AMPARs (Levchenko-Lambert et al., 2011). Therefore, to further validate that activation of KARs, rather than direct KA agonism of AMPARs, evokes the loss of surface AMPARs we also tested 1 μM KA, a concentration below the threshold for AMPAR activation (Dai et al., 2001) so inclusion of the AMPAR inhibitor GYKI 53655 was not required. In these, and subsequent experiments, we focused on surface expression of GluA2, which is routinely used as a reporter for AMPAR endocytosis (Evers et al., 2010; Nishimune et al., 1998). As for 10 μM KA stimulation, surface levels of GluA2, assessed by surface biotinylation followed by Western blotting, were significantly reduced by 1 μM KA application (Figure 1B).

### KA application decreases surface GluA2 in dendrites

**Figure 2:**
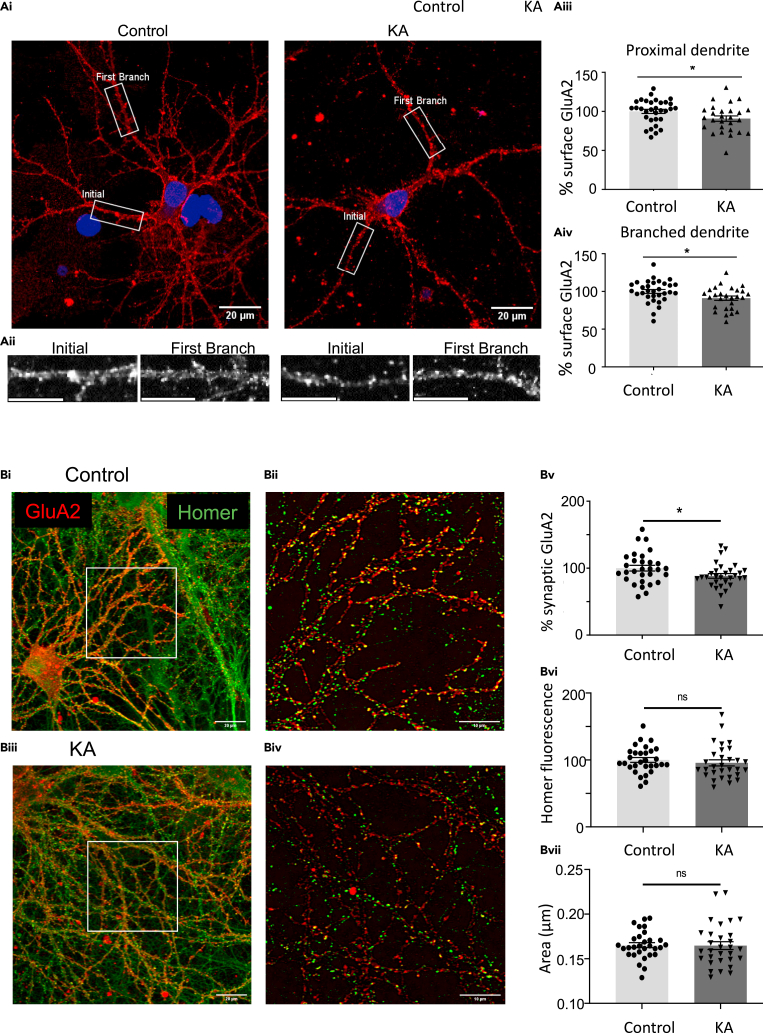
Imaging of DIV 18 hippocampal neurons shows a significant reduction in GluA2 surface expression after 10 μM KA for 20 min (A) Surface GluA2 in dendrites. (Ai) Representative images of control and KA-treated neurons showing proximal and first branch dendrites. Scale bar = 20 μm. (Aii) Expanded images of ROIs indicated in boxes in (Ai). Scale bar = 10 μm. (Aiii) Quantification of the intensity of surface GluA2 staining in proximal dendrites. (Aiv) Quantification of the intensity of surface GluA2 staining after the first dendritic branch. In all cases n = 32 cells, N = 3 independent dissections, ∗p < 0.05, Un-paired t test with Welch’s correction, error bars = SEM. (B) GluA2 surface expressed at synapses. Representative images of control and KA-treated neurons of surface GluA2 colocalised with the postsynaptic marker Homer. (Bii and Biv) are zooms of the ROI areas indicated in (Bi) and (Biii). Scale bars = 20 μm and 10 μm, respectively. (Bv) Quantification of the number of surface-expressed synaptic GluA2 ± KA treatment expressed as percentage of control. (Bvi) quantification of Homer (marker of synapse) fluorescence expressed as percentage of control ± KA treatment. (Bvi) area of Homer fluorescence ± KA treatment. In each condition n = 10 ROIs, each containing between 47 and 461 particles, were analyzed and averaged. Data from N = 3 independent dissections, ∗p < 0.05, Un-paired t test, error bars = SEM.

We next used live cell surface staining and fixed confocal imaging to monitor GluA2 surface expression in control and KA-treated hippocampal neurons. Neurons were pre-treated with 40 μM GYKI 53655 and 1 μM TTX for 30 min prior to application of 10 μM KA for 20 min, followed by live surface labeling of AMPARs with an N-terminal anti-GluA2 antibody. Consistent with the biochemical data, our imaging showed a significant reduction in the surface levels of GluA2 in segments of both proximal and branched secondary dendrites (Figure 2A).

### KA application decreases synaptic surface-expressed GluA2

To determine if the decrease in GluA2-containing AMPARs occurred at synapses, we monitored surface GluA2 expression specifically at puncta positive for the synaptic marker Homer (Hayashi et al., 2009; Tao-Cheng et al., 2014). As expected, the mean fluorescence of surface-expressed GluA2 specifically at Homer puncta was a significantly decreased (Figures 2Bi–Biv). Quantification of the data show that surface GluA2-containing AMPARs are decreased at synapses, but that the mean fluorescence and area of Homer puncta are not altered by KAR activation (Figures 2Bv–Bvii).

### KA-induced decreases in AMPAR surface expression are independent of NMDARs and mGluRs

**Figure 3:**
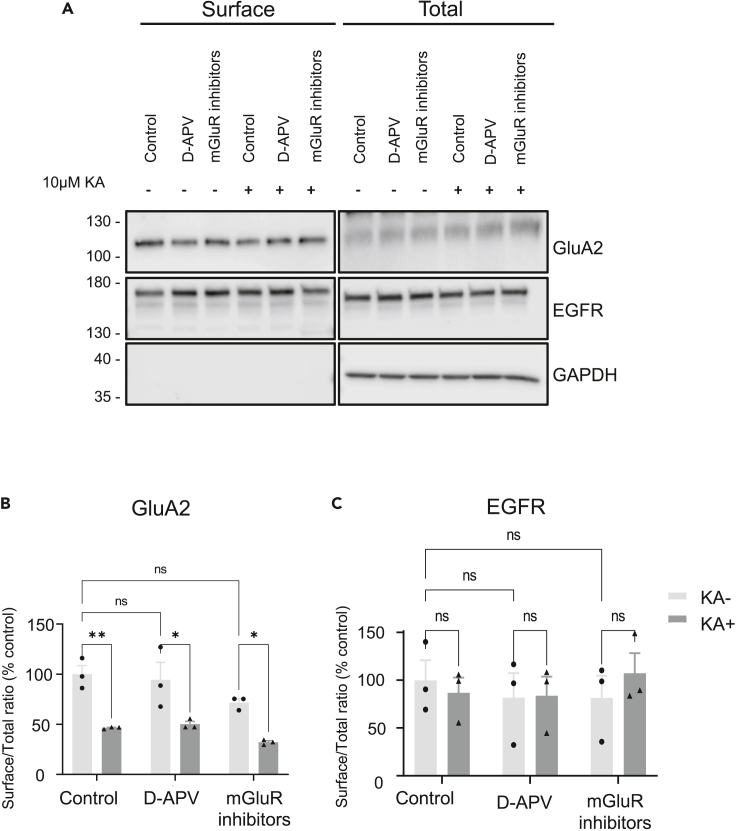
KA-induced decreases in AMPAR surface expression are independent of NMDAR or mGluR1/mGluR5 activation DIV18 hippocampal neurons were pre-treated for 30 min with 50 μM D-APV (NMDAR antagonist) or 10 μM MPEP (mGluR5 antagonist) along with 1 μM YM298198 (mGluR1 antagonist) in addition to 1 μM TTX and 40 μM GYKI53655. Neurons were then incubated for a further 20 min with vehicle or 10 μM KA. Surface proteins were biotinylated, isolated, and Western blotted. (A) Representative blot of surface and total levels of GluA2 and EGFR. GAPDH was used as a control to ensure no internal proteins were biotinylated. (B and C) Quantification of the surface to total ratio of GluA2 (B) and EGFR (C) expressed as a percentage of control. N = 3 independent dissections, ns p > 0.05, ∗p < 0.05, ∗∗p < 0.01; two-way ANOVA with Tukey’s multiple comparisons test, error bars = SEM.

To exclude any indirect effect mediated by either NMDARs or mGluRs, we pre-treated neurons with the NMDAR antagonist D-APV (50 μM) (Morris, 1989) or a combination of the mGluR5 antagonist (MPEP) (Lea and Faden, 2006) and the mGluR1 antagonist YM298198 (Kohara et al., 2005) (10 μM and 1 μM, respectively). As shown in Figure 3, 10 μM KA still caused a significant decrease in GluA2 surface expression in the presence of these drugs, whereas there was no change in surface EGFR levels under any condition, indicating the KA-induced decrease in surface GluA2 occurs in the absence of NMDAR and mGluR activity.

### GluK2 is required for the KA-evoked decrease in AMPAR surface expression

**Figure 4:**
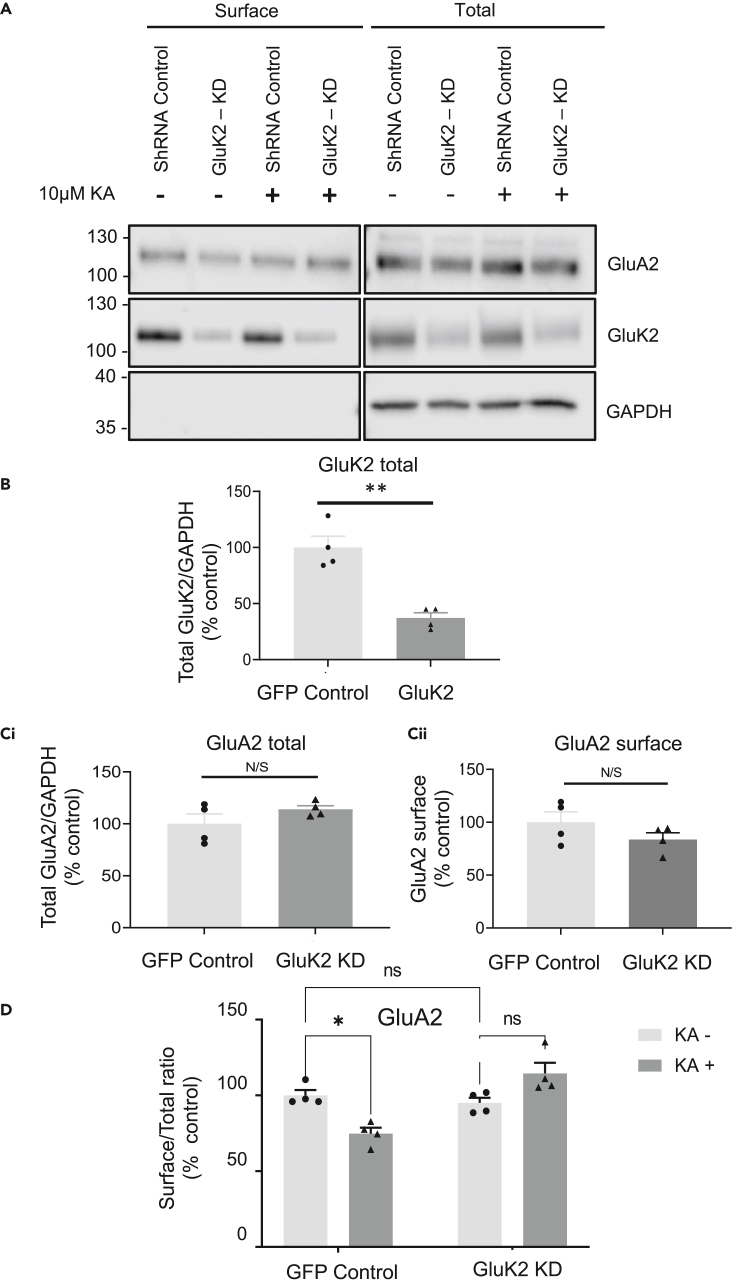
GluK2-containing KARs mediate the reduction in surface expression of AMPARs (A) DIV 10 hippocampal neurons were transduced with lentivirus expressing either control or GluK2 shRNA. After 7 days neurons were pre-treated for 30 min with 1 μM TTX and 40 μM GYKI 53655. Neurons were then treated with 10 μM KA or vehicle for another 20 min. Surface proteins were biotinylated, isolated, and Western blotted. Representative Western blots of surface and total levels of GluA2 and GluK2 (B) Total GluK2 levels following infection with GluK2 shRNA knockdown lentivirus. (C) Total (Ci) and surface (Cii) expressed GluA2 levels following GluK2 knockdown lentivirus. N = 4 independent dissections, ns = p > 0.05, ∗∗p < 0.01, Un-paired t test with Welch’s correction, error bars = SEM. (D) Quantification of the surface to total ratio of GluA2 expressed as a percentage of control. N = 4 independent dissections, ns = p > 0.05, ∗p < 0.05, ∗∗p < 0.01, two-way ANOVA with Tukey’s multiple comparisons test, error bars = SEM.

We have shown previously that the GluK2 KAR subunit is required for induction of KAR-LTP_AMPAR_ in the hippocampus (Petrovic et al., 2017). We therefore explored the role of GluK2-containing KARs in the KA-dependent reduction of surface GluA2 levels. Neurons were infected with lentiviruses either expressing a GluK2-targeting shRNA or a control virus expressing a non-targeting control shRNA. Consistent with our previous reports (Evans et al., 2017; Gurung et al., 2018), the GluK2 shRNA reduced total GluK2 levels by ∼70% (Figures 4A and 4B), but there was no significant difference in levels of total or surface GluA2 between control and GluK2 knockdown conditions (Figures 4A and 4C).

7 days post-transduction, neurons were pre-treated with 40 μM GYKI 53655 and 1 μM TTX for 30 min, prior to application of 10 μM KA for 20 min. Following the KA challenge neurons were subjected to surface biotinylation, streptavidin pulldown, and Western blotting (Figures 4A and 4D). As expected, in neurons expressing the control shRNA, KAR activation significantly decreased GluA2 surface expression. The KA-induced reduction in surface GluA2 did not occur in GluK2 knockdown neurons, indicating that the KA-mediated reduction in GluA2 surface expression requires the GluK2 KAR subunit.

### KA-evoked downregulation of surface AMPARs requires ionotropic KAR signaling

**Figure 5:**
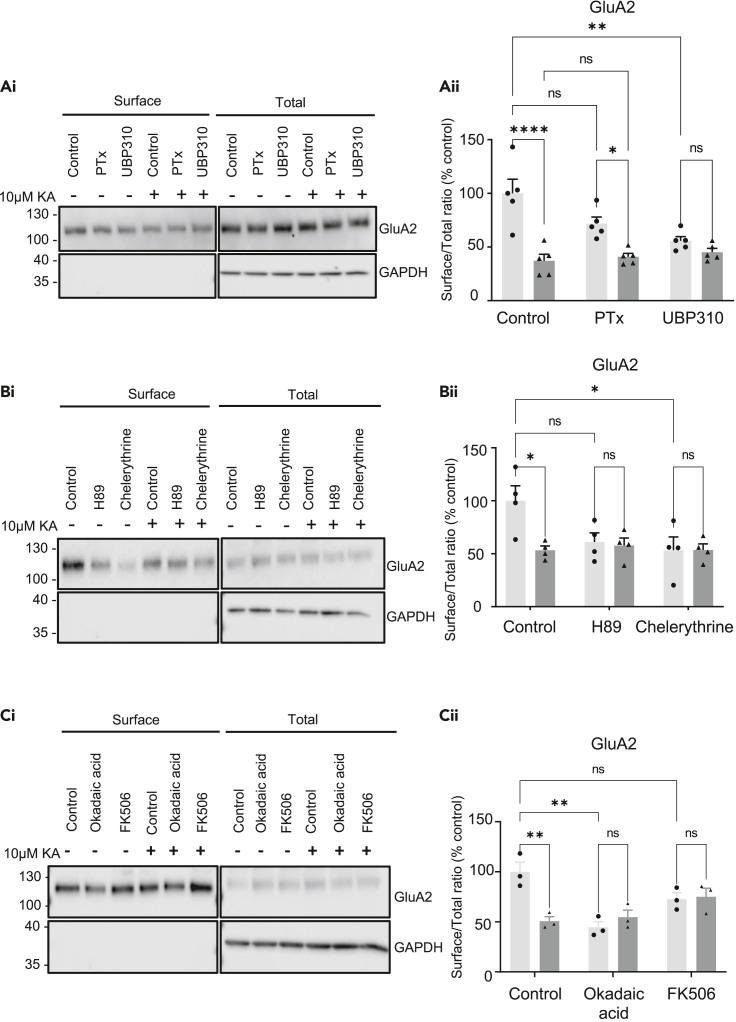
KA-induced decreases in AMPAR surface expression require ionotropic KAR signaling, PKA, and PKC (A) DIV 18 hippocampal neurons were pre-treated for 1 hr with 1 μg/mL PTx, or 30 min with 10 μM UBP 310 in addition to 1 μM TTX and 40 μM GYKI53655, then for a further 20 min with vehicle or 10 μM KA. Surface proteins were biotinylated, isolated, and Western blotted. (Ai) Representative blot of surface and total levels of GluA2. GAPDH was used as a control to ensure no internal proteins were biotinylated. (Aii) Surface to total ratio of GluA2. N = 5 independent dissections. See also Figure S1. (B) As (A), except neurons were pre-treated 40 μM GYKI 53655 and either 10 μM H89 or 5 μM Chelerythrine prior to 10 μM KA. (Bi) Representative blots of GluA2. (Bii) Quantification of the surface to total ratio of GluA2 expressed as a percentage of control. N = 4 independent dissections. (C) As (A), except neurons were pre-treated were pre-treated for 30 min with 1 μM Okadaic acid (PP1A and PP2A inhibitor) or 50 μM FK506 (calcineurin inhibitor) prior to 10 μM KA. (Ci) Representative blots of GluA2. (Cii) Quantification of the surface to total ratio of GluA2 expressed as a percentage of control. N = 3 independent dissections. In all cases ns p > 0.05, ∗p < 0.05, ∗∗p < 0.01, ∗∗∗p < 0.001, ∗∗∗p < 0.0001; two-way ANOVA with Tukey’s multiple comparisons test, error bars = SEM.

To investigate the signaling pathway required for the KAR-induced decrease in surface GluA2-containing AMPARs, we used the ionotropic KAR blocker UBP 310 (Dolman et al., 2005; Grosenbaugh et al., 2018; Petrovic et al., 2017; Pinheiro et al., 2013) and the Gα_i/o_ protein inhibitor pertussis toxin (PTx) (Petrovic et al., 2017). In contrast to KAR-LTP_AMPAR_, blocking KAR metabotropic signaling for 1 hr with 1 μg/mL PTx prior to KA stimulation did not prevent the KA-induced reduction in GluA2 surface expression (Figures 5A and S1). However, 10 μM UBP 310, an antagonist of postsynaptic ionotropic signaling through GluK2/GluK5-containing KARs did block the KA-evoked reduction in surface GluA2. Thus, our data indicate that KAR channel activity, but not G-protein mediated signaling, is required to initiate the downregulation of AMPAR surface expression. We note, however, that UBP 310 caused a significant reduction in surface GluA2 levels in the absence of KA stimulation, suggesting KAR activity may be required to maintain surface AMPAR expression. Consequently, the lack of KA-induced loss of surface GluA2 in the presence of UBP 310 may represent an occlusion effect.

### Effects of PKA and PKC on KA regulation of surface GluA2

Phosphorylation cascades play key roles in all identified forms of synaptic plasticity (Lu and Roche, 2012), and kinase activation is an integral downstream component of both ionotropic and metabotropic KAR signaling pathways (Evans et al., 2019). We therefore explored the role of the protein kinases PKA and PKC in modulating surface expression of GluA2-containing AMPARs in response to KA treatment because of their well-established roles in AMPAR trafficking and KAR signaling (Gonzalez-Gonzalez and Henley, 2013; Konopacki et al., 2011; Martin et al., 2008; Martin and Henley, 2004). AMPAR surface expression was assessed by surface biotinylation after 30 min pre-treatment of cultured hippocampal neurons with TTX (1 μM), GYKI 53655 (40 μM), and the PKA or PKC-specific inhibitors H89 (10 μM) or chelerythrine (5 μM), respectively. Neurons were then subjected to 20 min of sustained 10 μM KA stimulation. Both kinase inhibitors trended toward reduced basal surface levels of GluA2 suggesting that PKA/PKC signaling pathways support the surface expression of GluA2-containing AMPARs. We hypothesize that H89 and chelerythrine, like UBP 310, may lead to the loss of ‘removable’ AMPARs from the neuronal surface to a ‘floor level’ so that KA stimulation cannot decrease them any further. Thus, blocking PKA or PKC occludes the KA-induced reduction in GluA2 surface expression (Figure 5B).

In addition to kinases, the protein phosphatases PP1, PP2A, and PP2B (calcineurin) have been reported to be involved in forms of LTD (Belmeguenai and Hansel, 2005; Groth et al., 2003). We therefore investigated the effects of pre-treatment with inhibitors of these phosphatases (Figure 5C). We tested the effects of 1 μM okadaic acid to inhibit PP1 and PP2A (Schnabel et al., 2001), or 50 μM FK506 to inhibit calcineurin/PP2B (Beattie et al., 2000), on the KA-evoked decrease in GluA2 surface expression. Okadaic acid reduced surface levels of GluA2 in non-stimulated conditions, indicating roles for the protein phosphatases PP1/PP2A in regulating basal surface expression of AMPARs. These data suggest that okadaic acid occludes, whereas FK506 blocks the KA-evoked decrease in GluA2 surface expression.

Together, these data indicate that the KAR-induced decrease in surface GluA2-containing AMPARs does not occur in the absence of KAR channel opening. Moreover, blockade of PKC or PKA, which are activated downstream of ionotropic as well as metabotropic signaling, also reduces GluA2 surface expression and occludes the effect of KAR activation, demonstrating that PKA/PKC signaling support the surface targeting of GluA2. Interestingly, blocking dephosphorylation with phosphatase inhibitors also prevents the KAR-mediated decrease in GluA2 surface expression. The multiple phosphatase pathways involved in plasticity are complex (Foley et al., 2021), and our results indicate that both phosphorylation and dephosphorylation play key roles both in supplying surface AMPARs and allowing their removal in response to KA.

### KAR stimulation induces both short-term and long-term synaptic depression

**Figure 6:**
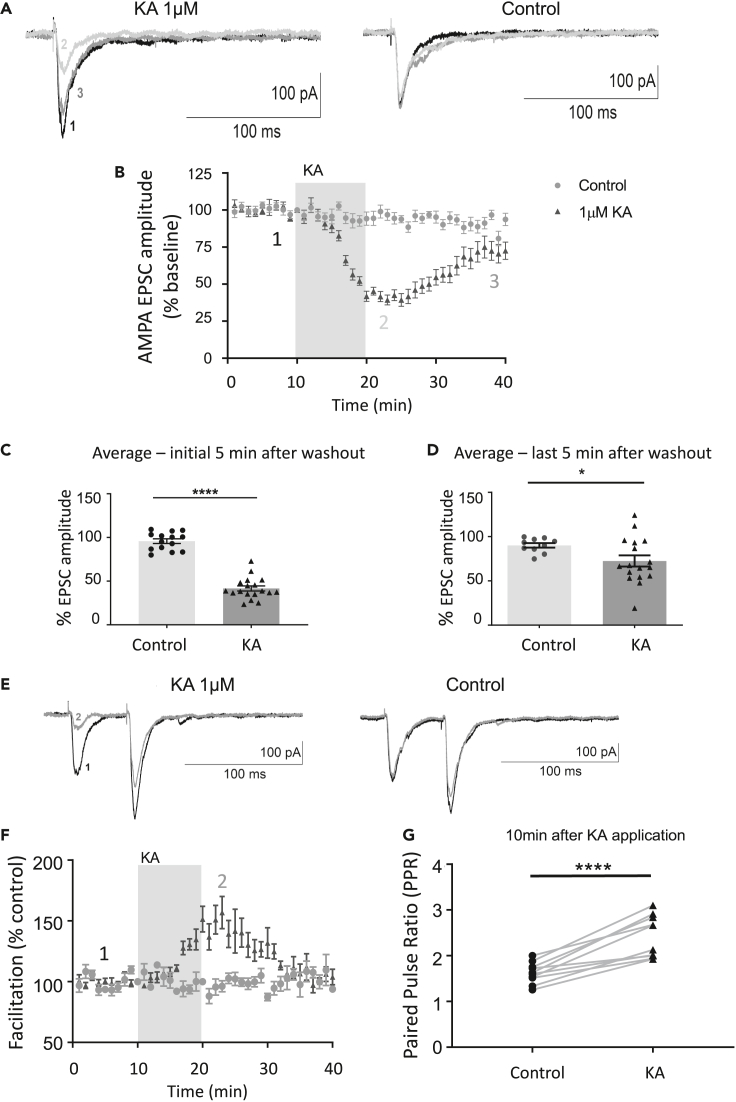
Sustained KAR activation induces depression of AMPAR EPSCs at hippocampal CA1 synapses (A) Example AMPAR EPSC traces in the presence or absence of 1 μM KA recorded at time points indicated in (B) in the CA1 region of acute hippocampal slices. (B) Plot of AMPAR EPSC amplitude. EPSCs were normalized to baseline corresponding to an initial 10 min prior to KA application. The traces were recorded in the presence of 50 μM D-AP5 and 50 μM picrotoxin. n = 14 and 18 cells for control and KA, respectively, from at least 4 different animals. (C and D) Quantification of mean AMPAR EPSC amplitudes in 5 min (C) and 20 min (D) after KA washout. Un-paired t test with Welch’s correction, error bars = SEM. n = 14 and 18 cells for control and KA, respectively, from at least 4 different animals. (E) Example AMPAR paired pulse EPSC traces in the presence or absence of 1 μM KA, recorded at time points indicated in (F). (F) Plot of paired pulse facilitation expressed as a percentage of control after 10 min of sustained stimulation with 1 μM KA. N = 10 cells from at least 4 different animals. Error bars = SEM. (G) Quantification of paired pulse ratio after 10 min of stimulation with 1 μM KA. N = 10 cells, ns = p > 0.05, ∗p < 0.05, ∗∗p < 0.01,∗∗∗p < 0.001, ∗∗∗∗p < 0.0001, paired t test.

We next investigated the effects of KA stimulation on synaptic function by monitoring AMPAR excitatory postsynaptic currents (EPSCs) in the CA1 region of acute rat hippocampal slices. Here, where key circuitry remains intact, we bath applied 1 μM KA for 10 min to avoid non-specific activation of AMPARs without the need for perfusion of GYKI 53655. Also included in the perfusate was 50 μM picrotoxin to prevent any confounding effect of KAR-induced GABA release from inhibitory interneurons and 50 μM D-AP5 to block NMDARs. Consistent with our biochemistry and imaging, AMPAR EPSCs were significantly reduced for 20 min after KA washout (Figure 6A-D). These results demonstrate that sustained KA application causes a long-term, NMDAR-independent reduction in synaptic AMPAR currents at CA1 synapses.

We also measured the paired pulse ratio (PPR) following KA stimulation to assess any changes in the probability of glutamate release (Debanne et al., 1996; Manabe et al., 1993). Immediately following KA stimulation, we observed an increase in PPR, indicating a decrease in the release probability characteristic of presynaptic short-term depression (Figure 6E-G) (Lauri et al., 2006). Importantly, however, the PPR returned to baseline levels within 10–20 min after KA washout, whereas AMPAR EPSCs remained depressed, suggesting the existence of a postsynaptic LTD of AMPAR transmission. We interpret these data to indicate that 1 μM KA for 10 min mediates both pre- and postsynaptically expressed depression of AMPAR EPSCs.

## Discussion

Both ionotropic and metabotropic KAR signaling pathways are important regulators of excitatory and inhibitory neurotransmission (Evans et al., 2019). We have shown previously that sustained KAR activation causes a PKC-dependent, PKA-independent, internalization of GluK2-containing KARs (Martin and Henley, 2004). Surprisingly, however, we observed that following an initial loss of surface KARs, a short kainate application elicited a long-lasting increase in surface-expressed KARs to levels significantly greater than those prior to the agonist challenge (Martin et al., 2008). We further showed that after initial endocytosis, transient agonist activation evokes increased KAR exocytosis via metabotropic signaling, which recruits Rab11-dependent, transferrin-positive endosomes to synapses and increases KAR recycling (Gonzalez-Gonzalez and Henley, 2013). Overall, these data suggest that KARs are subject to bidirectional regulation, allowing neurons to adapt their physiological responses to changes in synaptic activation.

These findings raised the question as to whether the KAR-initiated signaling was limited to the regulation of KARs themselves or whether KAR-mediated signaling also impacts on other synaptic proteins. Therefore, building on our KAR work we investigated if transient KAR activation also affected AMPARs. We demonstrated that both pharmacological and synaptic activation of KARs induced increased surface expression of AMPARs and evoked a KAR-dependent, NMDAR-independent, form of hippocampal LTP (KAR-LTP_AMPAR_) through a metabotropic signaling pathway (Petrovic et al., 2017).

In the current study, we show that prolonged KAR signaling can also downregulate AMPAR surface expression and evoke KAR-dependent, NMDAR-independent LTD (KAR-LTD_AMPAR_). Moreover, the KA-evoked reduction in surface AMPARs did not occur in GluK2 knock down neurons, indicating that GluK2-containing KARs mediate this effect. In contrast to KAR-LTP_AMPAR_, inhibition of metabotropic signaling did not prevent KAR-LTD_AMPAR_, whereas UBP 310, an antagonist of postsynaptic ionotropic signaling through GluK2/GluK5-containing KARs (Grosenbaugh et al., 2018; Petrovic et al., 2017; Pinheiro et al., 2013), did. The basal surface expression of GluA2 appeared to be reduced in the presence of metabotropic and/or ionotropic inhibitors, although this was not significant for PTx. It should be noted that PTx inhibits the signaling of multiple G protein-coupled receptors in addition to the metabotropic actions of KARs. The significant decrease in basal GluA2 surface levels in the presence of UBP 310 suggests KARs may also be required for maintaining tonic activity of AMPARs. Thus, through a yet unidentified pathway, UBP 310 can occlude LTD by decreasing the population of “endocysosable” AMPARs at the cell surface thereby diminishing the pool available for removal by KAR activation.

The mechanisms and roles of AMPAR subunit phosphorylation have been an area of intense study for many years (for reviews see Lu and Roche (2012) and Wang et al. (2014)). Phosphorylation of the GluA1 subunit, which contains phosphorylation sites for PKC and PKA, as well as sites for other kinases, has been most extensively investigated. The functional implications of AMPAR subunit phosphorylation have been questioned by one study which reported almost no phosphorylated GluA1 *in vivo* (Hosokawa et al., 2015). However, a subsequent study directly refuted that finding, showing that 12%–50% of the total population of GluA1 is phosphorylated under basal and stimulated conditions *in vitro* and *in vivo* (Diering et al., 2016). Moreover, it has been reported previously that activation of PKA with forskolin promotes surface trafficking of GluA1-containing AMPARs without affecting rates of endocytosis, resulting in increased AMPAR surface expression (Man et al., 2007). Our experiments using the PKA inhibitor H89 decreased AMPAR surface expression, consistent with reduced surface trafficking but unaffected endocytosis. Thus, similar to our data using UBP 310, we propose that a lack of ‘readily endocytosable’ AMPARs at the cell surface in the presence of H89 or chelerythrine occludes the KA-evoked reduction in surface AMPARs.

The GluA2 subunit contains a PKC site at Ser880 (Matsuda et al., 1999), located within the PDZ ligand at the extreme C-terminus, which selectively controls interactions with the PDZ domain containing protein GRIP1 (glutamate receptor-interacting protein) but not PICK1 (protein interacting with C kinase 1), which also binds the GluA2 PDZ ligand (Chung et al., 2000). It has been reported that PKC phosphorylation of Ser880 releases GRIP1, favoring PICK1 binding and leading to internalization of surface GluA2 (Chung et al., 2000). Moreover, a phosphomimetic mutant of Ser880 excludes GluA2-containing AMPARs from synapses, depresses transmission, and partially occludes LTD. Conversely, a phosphonull mutation at Ser880 reduces LTD (Seidenman et al., 2003). However, the situation is complex, and it has also been reported that GRIP, rather than holding AMPARs at the surface, stabilizes internalized receptors in an intracellular pool, and prevents them from recycling back to the plasma membrane or entering a degradative pathway (Braithwaite et al., 2002; Daw et al., 2000). Moreover, KAR endocytosis involves PKC activation, whereas PKC phosphorylation promotes surface expression of GluA1-containing AMPARs, and these effects have been linked to regulation of interactions of GluK2 and GluA1 subunits with the cytoskeletal adaptor protein 4.1N (Copits and Swanson, 2013; Shen et al., 2000).

Our data suggest that blocking PKC or PKA reduce GluA2-containing AMPAR surface expression and occlude KAR-evoked LTD suggesting tonic PKC and PKA activity are required to maintain surface AMPAR levels under control conditions. Moreover, our results raise the possibility that KAR activation potentially causes LTD by reducing PKC/PKA activity. However, it is important to note that our experiments blocking PKC and PKA do not specifically target GluA2, and the effects we observe could be mediated via altering phosphorylation of other core or auxiliary subunits in the AMPAR complex and/or by actions on interacting proteins. Interestingly, it has been reported recently that KAR activation causes a Ca^2+^- and PKC-dependent endocytosis of glycine receptors (GlyRs) and reduced GlyR-mediated synaptic activity (Sun et al., 2014). The proposed mechanism involves deSUMOylation of PKC and it is possible that an analogous PKC-deSUMOylation pathway may exist for the cross talk between KARs and AMPARs.

We also tested the effect of phosphatase inhibitor okadaic acid (PP1A and PP2A) and FK506 (calcineurin/PP2A). These too prevented the KA-evoked decrease in surface GluA2-containing AMPARs. Interestingly, okadaic acid reduced the basal levels of surface GluA2, indicating roles for the protein phosphatases PP1/PP2A in regulating basal surface expression of AMPARs. Together, we interpret these results to suggest that the dynamics of phosphorylation and dephosphorylation are clearly important both for maintaining the delivery of surface AMPARs and in their KAR-initiated removal.

The current consensus is that AMPAR surface expression and plasticity is mediated via phosphorylation (and other modifications) of the C-terminal domains of AMPAR subunits regulating the trapping or release of receptors at pre-existing “slots” in the postsynaptic density (PSD) (Hayashi et al., 2000; Henley and Wilkinson, 2016). However, in conditional knockout experiments in which GluA1, GluA2, and GluA3 subunits were ablated and replaced with either GluA1 or GluA2 subunits lacking a C-terminal domain, basal AMPAR trafficking and LTP were shown to be normal (Granger et al., 2013) (but see Zhou et al. (2018)). Most recently, a knock-in mouse study in which the C-terminal domain of endogenous GluA1 was truncated reported no deficit in basal synaptic transmission, LTP, or spatial memory (Diaz-Alonso et al., 2020). These authors propose an alternative model in which it is the number of slots rather than the C-terminal domains of the AMPAR subunits themselves that regulate the extent of AMPAR surface expression and plasticity at synapses (Diaz-Alonso et al., 2020). Thus, it is possible that phosphorylation is required to overcome a yet unidentified, negative modulatory effect, which is lacking in phosphodeficient AMPAR subunits. Overall, the currently available evidence suggests that plasticity involves the formation/unmasking or removal/masking of slots in the PSD to snare or release passively diffusing AMPARs, potentially irrespective of their phosphorylation or other posttranslational modifications (Diaz-Alonso et al., 2020).

In terms of our results, what then does this “slot availability” model predict? We suggest that constitutive PKC and PKA activity may be required to maintain slots, and thus blocking these kinases leads to a decrease in slot number, which may explain why blocking PKC and PKA occludes KAR-LTD_AMPAR_. We note, however, that our data demonstrate that PKA/PKC signaling is required to sustain surface GluA2 levels, and we do not directly address whether these kinases regulate incorporation of GluA2 into synaptic slots, although we do show that synaptic GluA2 levels are decreased by KA stimulation.

Consistent with these biochemical data, synaptic electrophysiology revealed two phases to the depression of AMPAR EPSC responses following KA activation and washout. The initial depression in EPSCs correlated with a rise in the PPR, which returned to baseline after 10 min of KA washout. However, after this initial short-term change in PPR returned to control levels, AMPAR EPSCs remained depressed, indicating a postsynaptic locus of KAR-LTD_AMPAR_. Thus, KA application reduces synaptic activity via an initial presynaptic mechanism which contributes to the early stages of depression in AMPAR EPSCs, but KAR-mediated loss of postsynaptic surface-expressed AMPARs maintains KAR-LTD_AMPAR_.

In summary, we show that in addition to transient KAR activation evoking KAR-LTP_AMPAR_, sustained KAR activation can induce KAR-LTD_AMPAR_. The underlying mechanisms appear to differ since KAR-LTP_AMPAR_ is mediated via a metabotropic signaling pathway, whereas KAR-LTD_AMPAR_ is blocked by the ionotropic KAR signaling blocker UBP 310. The physiological/pathological roles of KAR-mediated regulation of AMPAR surface expression at synapses and synaptic plasticity, and how they fit into the larger picture of NMDAR- and mGluR-mediated forms of plasticity remain to be elucidated. However, since KAR abundance and dysfunction is strongly linked to epilepsy (Crepel and Mulle, 2015; Peret et al., 2014), it is tempting to speculate that dysfunctional KAR-mediated plasticity of AMPARs, either directly or through affecting the availability of AMPAR slot proteins, could play important roles in neurological diseases.

### Limitations of the study

We are mindful that the pharmacological inhibition of metabotropic (pertussis toxin) or ionotropic (UBP 310) signaling through KARs, as well as the inhibition of PKC (chelerythrine), PKA (H89), PP1A and PP2A (okadaic acid), and calcineurin/PP2B (FK506), although selective for their targets, will have wider effects than on solely KAR signaling. Nonetheless, we contend that the key point is that these drugs have distinct effects on the KA-evoked changes in surface GluA2, strongly implicating their target proteins in the underpinning mechanisms of KAR-evoked LTD_AMPAR_. However, as noted above, inhibition of several of these pathways leads to a decrease in surface AMPARs under basal conditions, suggesting their blockade may occlude the KA-induced loss of surface GluA2. Potentially, these pathways could be involved in “priming” KAR-LTD_AMPAR_ by suppling removable AMPARs to the cell surface, rather than in the direct mechanism of KAR-mediated AMPAR removal. Further work will therefore be required to distinguish between these possibilities.

We believe the data we present provide compelling evidence that GluK2-containing KARs can mediate LTD of AMPARs in cultured neuronal systems. This work, however, is restricted to pharmacological approaches using the application of kainate to induce this form of plasticity. We have not yet identified the physiological/pathological conditions under which KAR-LTD_AMPAR_ occurs in intact hippocampal slices or *in vivo*. Experiments to define the precise physiological induction conditions, roles, and consequences of KAR-LTD_AMPAR_, and the relationship of this to other forms of LTD represent exciting avenues for future investigation.

## STAR★Methods

### Key resources table

**Table:** 

REAGENT or RESOURCE	SOURCE	IDENTIFIER
**Antibodies**		

Rabbit Polyclonal Anti-Glutamate receptor 1	Millipore	AB1504
Mouse Monoclonal Anti-Glutamate receptor 2, extracellular clone,6C4	Millipore	MAB397
Rabbit Monoclonal Anti-GluR6/7, clone NL9	Millipore	04-921
Mouse Monoclonal Anti-Glutamate Receptor	BD Pharmingen	556341
Rabbit Monoclonal Anti EGFR	Abcam	Ab52894
Mouse Monoclonal Anti GAPDH	Abcam	Ab8245
Donkey Anti-Mouse Cy3	Jackson immuno	715-165-150
Chicken Polyclonal Anti-Homer 1	Synaptic Systems	160 006

**Chemicals, Peptides, and Recombinant Proteins**		

Chelerythrine	Sigma-Aldrich	C2932, CAS Number: 3895-92-9
CNQX	Sigma-Aldrich	C127, CAS Number: 115066-14-3
D-AP5	Tocris	Catalog Number: 0106
Glycine	Sigma-Aldrich	G8790, CAS Number: 56-40-6
GYKI 53655	Tocris	Catalog Number:2555
H89	Tocris	Catalog Number: 2910
Kainate	Tocris	Catalog Number: 0222
NMDA	Tocris	Catalog Number: 0114
Pertussis Toxin (PTx)	Tocris	Catalog Number: 3097
TTX	Tocris	Catalog Number: 1069
UBP 310	Tocris	Catalog Number: 2079
Picrotoxin	Sigma-Aldrich	P1675, CAS Number124-87-8
EZ-Link^TM^ Sulfo-NHS-SS-Biotin	Thermo Fisher	Catalog Number: 21331
Streptavidin-Agarose from *Streptomyces Avidinii*	Sigma-Aldrich	S1638-5ML

**Experimental models: cell lines**		

Primary Rat Hippocampal Neuronal Cell cultures	(Fletcher-Jones et al., 2019; Konopacki et al., 2011).	N/A

**Experimental Models: Organisms/Strains**		

Wistar rat	University of Bristol Animal Services	N/A

**Recombinant DNA**		

GluK2 shRNA targeting: GCCGTTTATGACACTTGGA (pXLG3-GFP vector)	(Rocca et al., 2017)	N/A
Non-targeting shRNA: AATTCTCCGAACGTGTCAC (pXLG3-GFP vector)	(Rocca et al., 2017)	N/A

**Software and Algorithms**		

WinLTP v1.11 acquisition software	(Anderson and Collingridge, 2007)	https://www.winltp.com/
GraphPad Prism version 8.0	This paper	https://www.graphpad.com/scientific-software/prism/
LI-COR Biosciences ImageStudio Lite Version 5.2	(Evans et al., 2019)	https://www.licor.com/bio/image-studio-lite/download

### Resource availability

#### Lead contact

Further information and requests for resources and reagents should be directed to and will be fulfilled by the Lead Contact, Jeremy Henley (j.m.henley@bristol.ac.uk).

#### Materials availability

This study did not generate new unique reagents.

#### Dissociated primary neuronal culture

Pregnant E18 (Embryonic Day 18) Han Wistar rats were anesthetised with isoflurane and humanely sacrificed using schedule 1 method (cervical dislocation) after checking for reflexes. Brains of male and female embryos were removed under a dissection microscope, and the hippocampi excised for hippocampal cultures and the rest of the cortex was used for preparation of cortical cultures.

Dissected hippocampi and cortices were transferred to 15ml and 50ml falcon tubes respectively under a laminar airflow chamber and cortices were further dissociated using a sterile scalpel blade. Tissues were washed three times with 10ml HBSS for hippocampi and 30ml for cortices. Cortices were incubated for 15 mins in 30ml HBSS containing 0.005% trypsin/EDTA and hippocampi for 9 mins in 30ml HBSS containing 0.005% trypsin/EDTA at 37°C with frequent inverting. The trypsinised tissues were washed three times with HBSS and one wash with 1ml plating media for hippocampi and 5ml plating media for cortices. The cell suspensions were triturated with 1ml pipette for hippocampus and 5ml serological pipette for cortical tissues. Hippocampal and cortical cell suspension were diluted up to 4ml and 20ml respectively with plating media.

The cells were counted in a hemocytometer. For biochemistry experiments, 500,000 cells per well and for imagining 110,000 cells per well were plated in pre-treated 6 well tissue culture dish containing plating media and incubated in a 37°C incubator. 2 hrs after seeding the cells, the plating media was replaced with 2ml of feeding media (Neurobasal® medium supplemented with 2% B27 and 1% glutamax) and replaced in a 37°C incubator. 7 days later the neurones were fed with a further 1ml of feeding media and stored until use.

#### Acute hippocampal slice preparation from postnatal male and female Han Wistar rats for electrophysiology

Postnatal day 13-15 male and female Han Wistar rats were anaesthetized with 4% isoflurane and decapitated. Brains were rapidly removed and placed in 4°C oxygenated (95% O_2_, 5% CO_2_) sucrose solution (in mM: sucrose 189, D-glucose 10, NaHCO_3_ 26, KCl 3, MgSO_4_ 5, CaCl_2_ 0.1 and NaH_2_PO_4_ 1.25). Parasagittal hippocampal slices 400μm thick were prepared using a vibratome (7000smz-2, Campden Instruments). Slices were kept in a slices holder containing artificial cerebrospinal fluid (aCSF in mM): NaCl 124, NaHCO_3_ 26, KCl 3, NaH_2_PO_4_ 1, MgSO_4_ 1, D-glucose 10 and CaCl_2_ 2; and incubated for 30 mins at 35°C and then for a further 30 mins at room temperature before use.

### Method details

#### Lentivirus production and transduction

DMEM was filter sterilized using a 0.2μM syringe filter and 2.5ml of plain DMEM was mixed with 20μl of XLG viral vector consisting of shRNA expressed under H1 promoter (pXLG3-100bp stuffer), 5μg pDMD2.G packaging vector (Addgene) and 15μg of p8.91 helper vector (Addgene). The shRNA target sequences were Control, non-targeting shRNA: AATTCTCCGAACGTGTCAC; GluK2-targeting shRNA: GCCGTTTATGACACTTGGA. 2.5ml of plain DMEM media was mixed with 4.8% 1mg/ml polyethylenimide (PEI) in a sterile 15ml falcon tube. The mixture was mixed thoroughly, and filter sterilized into a fresh 15ml falcon tube with a 0.2μM syringe filter. This was left at RT for 2-3 mins with occasional mixing. The transfection media was prepared by mixing PEI-DMEM with the DNA mixture. Culture plates containing HEK293T cells were washed twice with 6ml of plain media and 5ml of transfection media was slowly added and left for 4 hours at 37°C. After incubation, the transfection media was aspirated and supplied with 7ml of pre-warmed neuronal feeding media or DMEM. The cells were placed back in the incubator for 2-3 days to produce virus. Media containing the virus was transferred into a fresh 15ml falcon tube and centrifuged at to 3000g for 10 minutes at 4°C to pellet cellular debris. The virus containing supernatant was syringe filtered (0.45μM) into a fresh 15ml falcon, aliquoted and stored at -80°C until further use.

#### Sustained KA stimulation

Hippocampal neurons were plated in a 6 well culture dish (500,000 cells per well) and maintained in a 37°C incubator. On DIV 17-18, the neurons were pre-treated with 1μM TTX (Tocris) and GYKI 53655 (Tocris), with or without additional drugs in Earle’s Buffer (140mM NaCl, 5mM KCl, 1.8mM CaCl_2_, 0.8mM MgCl_2_, 25mM HEPES, 0.9g/L D-Glucose, pH 7.4) and placed back in the incubator for 30 mins. After the incubation, 10μM KA was added to the wells and incubated for 20 mins at 37°C.

#### Neuronal surface biotinylation

After KA stimulation, neurones were cooled down to 4°C post treatment to prevent further trafficking of receptors. The wells were washed twice with 2ml of cold Earle’s Buffer (EBS) (140mM NaCl, 5mM KCl, 1.8mM CaCl_2_, 0.8mM MgCl_2_, 25mM HEPES and 0.9g/L D-glucose). The surface proteins were tagged with 1.5ml of Sulfo-NHS-SS-Biotin (0.3mg/ml diluted in 1X EBSS) (Thermo Fisher, Cat No.21331), for 10 mins with gentle movement every 2 mins and washed three times with 2ml of EBSS. 2ml of 100mM NH_4_Cl was added to scavenge the free biotin for 1 min. The cells were washed with 2ml of EBSS and were lysed in 200 μL lysis buffer (50mM Tris pH 7.4, 150mM NaCl, 1% triton X-100, 0.1% SDS, 1X protease inhibitor in ddH_2_O), scraped and transferred to a 1.5ml eppendorf. The cells were sonicated briefly with 3 pulses and were incubated on ice for 30 mins. After incubation cells were centrifuged at 20,000g at 4°C for 30 mins to get rid of cell debris.

The surface proteins were pulled down with 150μl Pierce^TM^ Streptavidin UltraLink^TM^ resin beads. The beads were washed twice with Lysis buffer devoid of protease inhibitor cocktail by centrifuging at 1500g at 4°C for 2 mins followed by aspirating the buffer containing supernatant. 80μl of lysate was added to the beads and mixed on a rotating wheel at 4°C for 1 hr. Following incubation, the beads were washed three times in lysis buffer and the supernatant was aspirated. The beads were re-suspended in 2X sample buffer and were heated at 95°C for 10 min on a heating block before western blotting.

#### Immunocytochemistry

Cells were incubated for 20 min in the primary antibody at RT. GluA2 N-Terminal Antibody (MAB397, Millipore, 1:70) was mixed in a 1.5mL Eppendorf containing 100μL of conditioned media (per coverslip). 90μL of the antibody containing media pipetted onto parafilm and the coverslips were gently placed with cells facing down on to the primary antibody. After incubation, the coverslips were placed back into a 6 well plate containing 2ml of DPBS (cells side facing up). The coverslips were washed 3-5 times in DPBS and fixed with 1ml of pre-warmed 4% formaldehyde + 5% sucrose for 12 mins. The cells were again washed 3 times in DPBS followed by a wash with 1ml of 100mM glycine dissolved in DPBS to quench residual formaldehyde. To remove glycine, the coverslips were again washed 3 times with DPBS. The cells were permeabilised and blocked using 3% BSA in DPBS containing 0.1% Triton-X 100 for 20 mins with gentle shaking at RT. Anti-mouse Cy3 antibody (Jackson ImmunoResearch, 1:400) secondary antibodies were diluted 1:400 in 3% BSA in 1XDPBS and were incubated for 1 hr (same as primary antibody incubation). After the incubation, the coverslips were placed back into the wells and were washed 3 times with DPBS. 40μL of mounting media (Fluoromount-GTM with DAPI (Thermo Fisher)) was pipetted on to slides and the coverslips were mounted (cells facing down) after gently dipping into ddH2O (to prevent salt crystal formation). The slides were left overnight to dry before imaging or storage at 4°C.

To measure the dendritic GluA2 distribution, random but clearly selectable and isolated dendrites were selected from initial dendrites (starting after cell body to first branch point) and first branch dendrites (from initial branch point to the subsequent branch point). For both categories, at least 4 dendrites were selected, and the branches were traced using the ‘Simple Neurite Tracer’ tool, filled automatically to the thickness of the neurite to create masks of the dendritic structure within the Region of Interest (ROI). The mean values of surface GluA2 intensity were measured from the masks by using Fiji software and the intensity values were averaged for each ROI of each neuron and analyzed in Graph Prism version 9.1.2.

For assessment of synaptic GluA2, cells were additionally stained for the postsynaptic marker Homer (anti-Homer, Synaptic Systems, 1:500) for 1 hr at RT following fixation and permeabilisation. Confocal imaging was carried out on a Leica SP8 with a 100x objective, 2x zoom, 10 ROIs per condition. Homer-stained ROIs were background subtracted, filtered (median), and auto thresholded (Otsu) in FIJI. Particles 20 pixels or larger were analyzed for mean GluA2 and Homer fluorescence. For each ROI, between 47 and 461 particles were analyzed and averaged together. 10 ROIs per independent experiment, 3 independent dissections.

#### Electrophysiology

Hippocampal slices were placed in a submerged holding chamber and perfused with 30°C oxygenated aCSF at 2ml min^-1^. Excitatory postsynaptic currents (EPSCs) of AMPA transmission were evoked at -70mV by stimulating the Schaffer collateral pathway and recorded from CA1 pyramidal neurons. Pyramidal neurons were patch-clamped in the whole-cell configuration using borosilicate glass (Harvard Apparatus) electrodes with a resistance of 2-5 MΩ and were backfilled with a solution containing (in mM): CsMeSO_4_ 130, NaCl 8, Mg-ATP 4, Na-GTP 0.3, EGTA 0.5, HEPES 10, QX-314-Cl 5; pH 7.2. The CA3 area of the hippocampal slices was removed using a scalpel blade in order to minimize epileptic activity. D-AP5 (50μM) and picrotoxin (50μM) were bath applied to isolate AMPA-mediated EPSCs. Cells in which the series resistance changed above 20 MΩ or deviated by 20% were discarded.

After a 10 min stable baseline was achieved, 1μM kainic acid was bath applied for 10 mins followed by a 30 min washout period.

Signals were low-pass filtered at 2 kHz and digitized at 10 kHz using a Axopatch 200B amplifier (Molecular Devices) and WinLTP v1.11 acquisition software (Anderson and Collingridge, 2007).

### Quantification and statistical analysis

For each experiment, the signal for each condition was divided by the mean overall signal from that experiment. This analysis was performed for each replicate experiment, and for presentation purposes, the mean of the control condition set to 100.

All graphs were generated, and statistical tests performed, using GraphPad Prism version 9.1.2. Our sample sizes correspond to previous published results and no statistical tests were performed to predetermine the sample size (Gonzalez-Gonzalez and Henley, 2013; Petrovic et al., 2017). The details of the statistical tests performed on each experiment are explained in the figure legend along with p-values and error bars. Number of cells = n and number of independent dissections/number of animals = N.

## Data Availability

• **Data**: All data reported in this paper will be shared by the lead contact upon request. • **Code**: This paper does not report original code. • **Experimental model and subject details**: The following animals were obtained from animal services facility at the University of Bristol. All the animal experiments and procedures were performed in compliance with the UK Animal Scientific Procedures act (1986) and were guided by the Home Office Licensing Team at the University of Bristol. All animal procedures relating to this study were approved by the Animal Welfare and Ethics Review Board at the University of Bristol.
