# Supplementary material for: Sustained postsynaptic kainate receptor activation downregulates AMPA receptor surface expression and induces hippocampal LTD
Source: iScience. 2021 Aug 25;24(9):103029. doi: 10.1016/j.isci.2021.103029 (PMC8441151; doi:10.1016/j.isci.2021.103029)
Supplement: Document S1. Figure S1 [file mmc1.pdf]

iScience, Volume *24*

Supplemental information

Sustained postsynaptic kainate receptor activation downregulates AMPA receptor surface expression and induces hippocampal LTD

Jithin D. Nair, Ellen Braksator, Busra P. Yucel, Alexandra Fletcher-Jones, Richard Seager, Jack R. Mellor, Zafar I. Bashir, Kevin A. Wilkinson, and Jeremy M. Henley

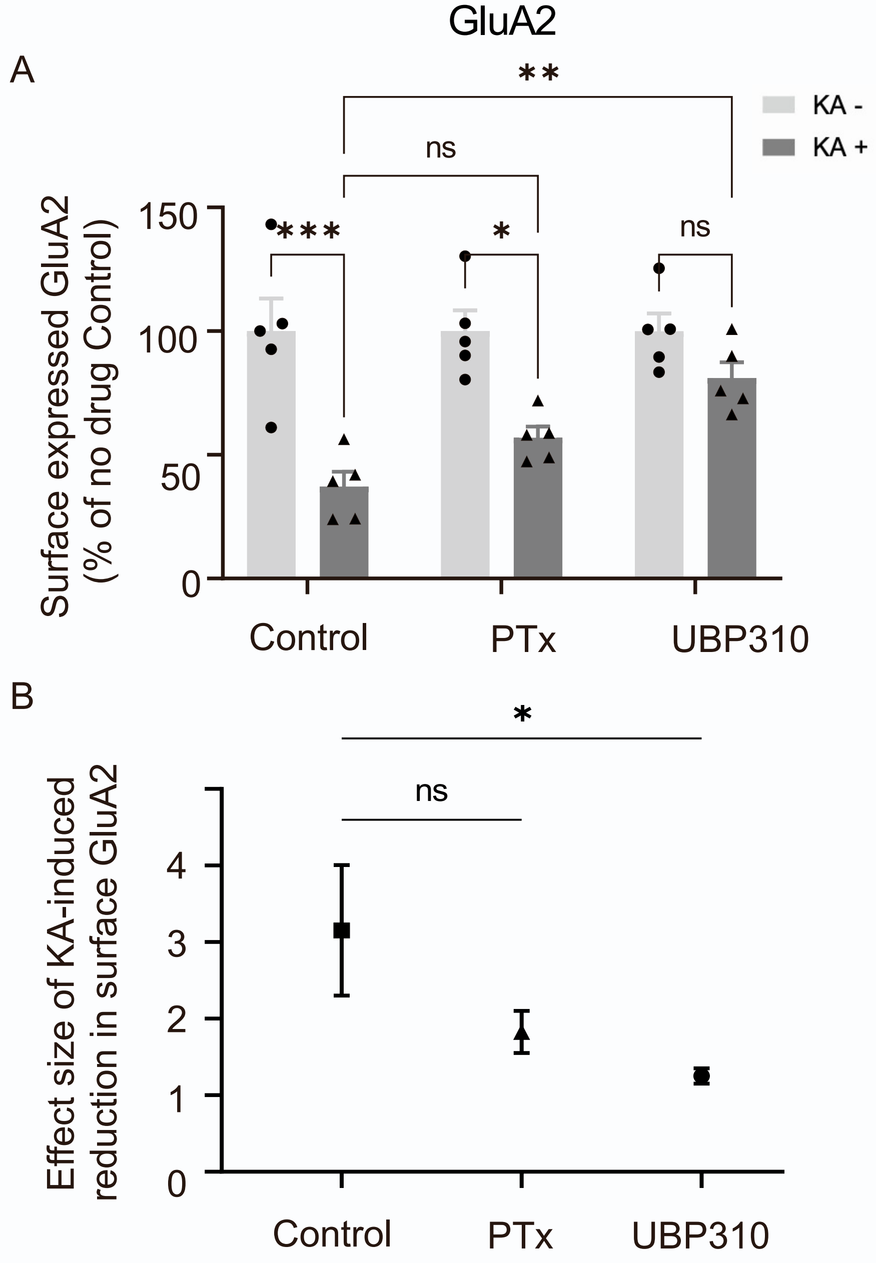

Figure S1 (relates to main text Figure 5) Effect size of KA-induced reduction in surface GluA2 under control, PTx and USB310 conditions

A) Quantification of the surface to total ratio of GluA2 with Control, PTx and UBP310 without KA taken as 100%. N=4 experiments from independent dissections, ns = p>0.05, * p<0.05, **p<0.01, ***p<0.001. Two-way ANOVA with Tukey's multiple comparison test, error bar = SEM.

B) Effect size of KA-induced reduction in surface GluA2. N=5 experiments from independent dissections, ns=p>0.05, *p<0.05. One-way ANOVA with Dunnett's multiple comparison test, error bar = SEM.
